# Neighborhood Greenspace in Midlife and Late-Life Brain Imaging Outcomes: Multi-Ethnic Study of Atherosclerosis

**DOI:** 10.1093/geroni/igaf122.946

**Published:** 2025-12-31

**Authors:** Lilah Besser, Jana Hirsch, Susan Heckbert, Peter James, Marcia Pescador Jimenez, Kari Moore, Joel Kaufman, Timothy Hughes

**Affiliations:** University of Miami, Boca Raton, Florida, United States; Drexel University, Philadelphia, Pennsylvania, United States; University of Washington, Seattle, Washington, United States; University of California, Davis, Davis, California, United States; Boston University, Boston, Massachusetts, United States; Drexel University Dornsife School of Public Health, Philadelphia, Pennsylvania, United States; University of Washington, Seattle, Washington, United States; Wake Forest University School of Medicine, Winston-Salem, North Carolina, United States

## Abstract

We investigated associations between residential neighborhood greenspace in midlife and late-life magnetic resonance imaging (MRI) outcomes. We used data on 1,141 participants without dementia in the Multi-Ethnic Study of Atherosclerosis (MESA), restricting to those with address history covering midlife. Geocoded addresses (1980-2009) were used to derive midlife residential greenness using annual (summer:∼July 1) normalized difference vegetation index (NDVI) values from LANDSAT satellite imagery (range=0-1, closer to 1=greener) and annual values of % tree canopy and open/park space linearly interpolated from 2001-2011 National Land Cover Datasets. We calculated 10-year mean neighborhood greenspace values spanning midlife (45-54 years). MRI measures included hippocampal and white matter hyperintensity volumes and total gray matter volume (GMV) in Alzheimer’s disease (AD) prone regions of interest (ROI), which accounted for head size differences (e.g., (hippocampal volume/intracranial volume) x 100%). Multivariable linear regression estimated associations between midlife 10-year mean greenspace measures and later-life MRI outcomes, controlling for site, demographics (e.g., age, ethnoracial group, gender, education), neighborhood socioeconomic status, and comorbidities (e.g., hypertension, diabetes), measured at the time of MRI. Participants were 70.1±6 years old at MRI and 54% were women. Greater midlife residential greenness and % open/park space (but not % tree canopy) were associated with greater late-life GMV (%) in AD ROI (estimates per interquartile range (IQR), respectively=0.09, 95% CI = 0.04-0.14; estimate=0.09, 95% CI = 0.05-0.12). In a diverse US cohort, individuals living in greener neighborhoods in midlife exhibited greater late-life GMV (%) in AD ROI, possibly leading to a small reduction in AD risk that should be explored further.

